# Development of electron source feedback for enhanced X-ray beam stability at NSLS-II

**DOI:** 10.1107/S160057752600233X

**Published:** 2026-04-02

**Authors:** Sukho Kongtawong, Yoshiteru Hidaka, Hanfei Yan, Guimei Wang, Kiman Ha, Yuke Tian, Joseph Mead, Danny Padrazo, Yong Chu, Timur Shaftan

**Affiliations:** a National Synchrotron Light Source II (NSLS-II) Brookhaven National Laboratory,Upton NY USA; b Synchrotron Light Research Institute Nakhon Ratchasima Thailand; Brazilian Synchrotron Light Laboratory, Brazil

**Keywords:** source feedback, X-ray beam stability, X-ray nanoprobe, NSLS-II

## Abstract

Future synchrotron light sources are being designed to deliver significantly higher brightness and coherence, enabling faster scanning and more advanced imaging techniques. However, realizing these capabilities requires improved X-ray beam stability, as vibrations directly limit measurement accuracy and image quality. At the NSLS-II Hard X-ray Nanoprobe (HXN), a local mechanical feedback system mitigates low-frequency motion but provides only limited suppression of dominant vibrations at 27 Hz and 120 Hz. To address this limitation, this work explores the use of the electron beam itself as the actuator for feedback by integrating X-ray beam position monitor signals into the fast orbit feedback system through a dedicated electrometer. Experimental results show that this approach effectively suppresses the dominant vibration peaks and enhances stability at the focusing point. Benchmark ptychography measurements further confirm that electron-beam-based feedback reduces background fluctuations and mitigates vibration-induced artifacts under fast-scanning conditions. These results establish the feasibility of using the electron beam as a feedback actuator for next-generation synchrotron applications and provide an experimental assessment of its potential and limitations.

## Abstract 2

An electron-source feedback method has been demonstrated at the NSLS-II Hard X-ray Nanoprobe, using X-ray beam position data to drive electron beam corrections. The approach suppresses dominant vibration peaks and significantly improves X-ray beam stability and ptychographic image quality.

## 1. Introduction

Future synchrotron light sources worldwide are being upgraded or newly constructed to deliver higher brightness and coherence. The planned upgrade to NSLS-IIU at the National Synchrotron Light Source II (NSLS-II) targets emittance values as low as 23.4 pm (3 GeV) and 42.5 pm (4 GeV), leading to a substantial improvement in brightness (Song & Shaftan, 2024*a* ▸; Song & Shaftan, 2024*b* ▸). Such improvements directly benefit advanced imaging techniques that require a coherent beam, significantly reducing acquisition time and providing the time resolution needed for *in situ*/*operando* studies.

The Hard X-ray Nanoprobe (HXN) beamline at NSLS-II offers fly scan capability with scanning speeds in the kHz range (Xu *et al.*, 2024 ▸; Yan *et al.*, 2018 ▸). However, as scanning frequencies increase, artifacts from beam instability emerge, introducing background fluctuations that degrade measurement accuracy for weak-contrast features. At HXN, a general criterion for beam stability is that beam motion remains below approximately 10% of the beam size.

At the HXN beamline, a mechanical local feedback system suppresses X-ray beam motion by controlling the beamline optics. While effective at mitigating low-frequency disturbances, the mechanical feedback is less effective against dominant higher-frequency vibrations of the optics. To address this limitation, we demonstrate a method that uses the electron beam itself as the actuator to compensate for downstream X-ray vibrations. By leveraging the fast orbit feedback (FOFB) system’s bandwidth of approximately 400 Hz (Kongtawong *et al.*, 2020 ▸; Kongtawong *et al.*, 2021 ▸), this approach can mitigate vibrations that are beyond the reach of mechanical feedback.

The use of X-ray or photon beam position information to control the electron beam has been explored in a variety of contexts at synchrotron light sources. In most cases, these approaches have been developed for low-bandwidth feedback applications, targeting long-term drift and low-frequency beam motion rather than fast disturbances.

Early work at the Advanced Photon Source demonstrated the feasibility of using X-ray beam position monitors (XBPMs) to assist electron orbit correction in slow or direct current (DC) regimes (Singh *et al.*, 2000 ▸; Decker & Singh, 2001 ▸). These studies established the fundamental concept of photon-assisted orbit feedback but were not intended for high-bandwidth operation.

More recently, photon-assisted orbit feedback has been implemented at several synchrotron facilities worldwide, including SOLEIL, ALBA, PLS-II, and NSLS-II. At SOLEIL, bending-magnet photon beam position monitors were incorporated as additional sensors in the slow orbit feedback loop to improve photon source-point stability, while the fast orbit feedback continued to rely on electron beam position monitors (BPMs) (Hubert *et al.*, 2013 ▸). At ALBA, an X-ray BPM installed at a front-end was included in the vertical slow orbit feedback loop to improve photon source stability after injection (Marcos & Muñoz, 2013 ▸). At PLS-II, a photon BPM feedback scheme was developed in which local electron BPM offsets are updated on a time scale of seconds and corrected through the existing slow orbit feedback to maintain the photon beam position at the monitor location (Ko *et al.*, 2016 ▸). At NSLS-II, long-term orbit stabilization has been extended to a unified orbit feedback framework that supports local bumps incorporating X-ray BPMs, either alone or in combination with RF BPMs (Hidaka *et al.*, 2019 ▸; Hidaka *et al.*, 2022 ▸). In all of these implementations, photon or X-ray diagnostics are used in low-bandwidth feedback loops operating alongside the storage-ring electron beam orbit feedback.

In parallel with orbit-based approaches, dedicated feedback systems have also been developed at nanofocusing beamlines using X-ray diagnostics to directly control optical elements. For example, at the hard X-ray nanoprobe beamline of the Shanghai Synchrotron Radiation Facility, a dual-frequency feedback system was implemented using X-ray BPM signals to drive monochromator and mirror actuators, achieving improved beam position and energy stability through optics-based correction (Jiang *et al.*, 2025 ▸). Related approaches using optical and X-ray diagnostics to stabilize nanofocused beams via beamline components have also been demonstrated at other facilities (Tolentino *et al.*, 2023 ▸).

Fast X-ray feedback has been demonstrated as a special case at Diamond Light Source through an independent local feedback loop known as SOFFOX (Bloomer *et al.*, 2019 ▸). This system uses XBPM signals to sense transverse X-ray beam motion downstream of the monochromator and applies corrective electron beam orbit adjustments using a closed local bump generated by fast corrector magnets. The feedback is implemented as a dedicated beamline-level controller that operates alongside the storage-ring orbit feedback and is intended to correct the AC component of the X-ray beam motion. The SOFFOX system demonstrates the feasibility of using X-ray diagnostics to drive electron beam corrections in a fast feedback configuration.

Our implementation integrates X-ray position data directly into the global orbit feedback system through dedicated hardware, using the electron beam itself as the actuator. This approach extends a unified feedback architecture, previously limited to high-frequency electron-beam and low-frequency X-ray operation at several facilities, to higher feedback rates characteristic of fast X-ray feedback systems. By embedding X-ray-based correction within the global FOFB framework, this unified high-rate architecture has the potential to reduce competition between separate local and global control loops, thereby lowering the risk of loop interaction and oscillatory behavior while enabling suppression of higher-frequency X-ray beam motion.

In this work, we present the concept, stability analysis, implementation, and experimental demonstration of an electron-based compensation method. Some preliminary aspects of this approach have been previously published (Kongtawong *et al.*, 2025 ▸). Experimentally, comparisons of recent ptychographic reconstructions with and without source feedback show that this method markedly enhances X-ray beam stability, achieving levels of vibration suppression not attainable with conventional mechanical feedback. This paper highlights both the potential and the challenges of integrating this method into existing feedback systems to meet the beam stability requirements of next-generation synchrotron experiments.

## 2. Feedback system and beam stability

### 2.1. HXN layout and feedback architecture

**Figure 1:**
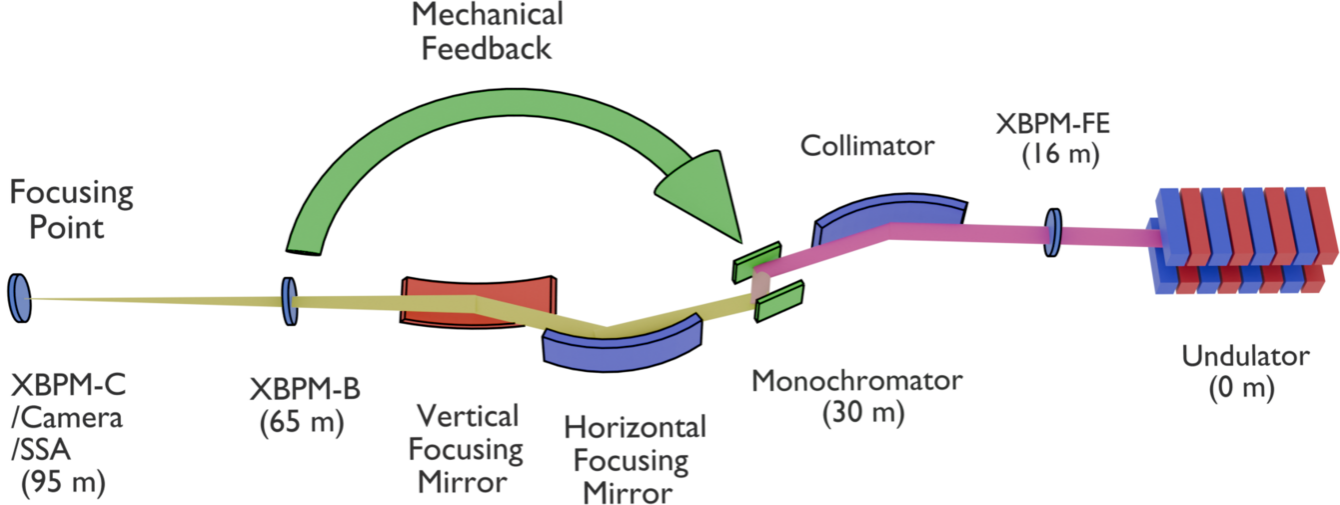
Schematic layout of the HXN beamline showing the XBPMs and camera (not to scale).

HXN implements a local mechanical feedback system to stabilize X-ray beam vibrations. Fig. 1 ▸ shows the layout of the HXN beamline relevant to feedback and beam position monitoring. Three XBPMs are installed along the beamline: one at the front-end (XBPM-FE), 16 m from the electron source; XBPM-B in Hutch B, 65 m from the source; and XBPM-C in Hutch C, located at the focusing point of the beam and the secondary source aperture (SSA), about 95 m from the source. At this focus, a camera provides complementary imaging of the X-ray beam to cross-check the XBPM signals. At the HXN beamline, XBPM-B employs a quadrant-electrode diamond detector, while XBPM-C uses a resistive diamond position-sensitive detector (Ilinski, 2018 ▸; Desjardins *et al.*, 2018 ▸). The XBPM signals are acquired using an electrometer developed in house, streamed through an EPICS IOC at 100 Hz in normal operation, and used to compute correction signals applied to the monochromator piezo actuator.

As shown in Fig. 1 ▸, the mechanical feedback system reads the X-ray beam position from XBPM-B and calculates the required correction. The controller then sends a signal to the monochromator in Hutch A, which houses the optical elements of the beamline, such as the collimator, monochromator, and focusing mirrors. By adjusting the pitch and roll of the monochromator, the system corrects horizontal and vertical beam positions, respectively, thereby stabilizing the beam at XBPM-B. This feedback has been implemented into HXN operations and improves low-frequency beam stability.

### 2.2. Beam stability characterization

**Figure 2:**
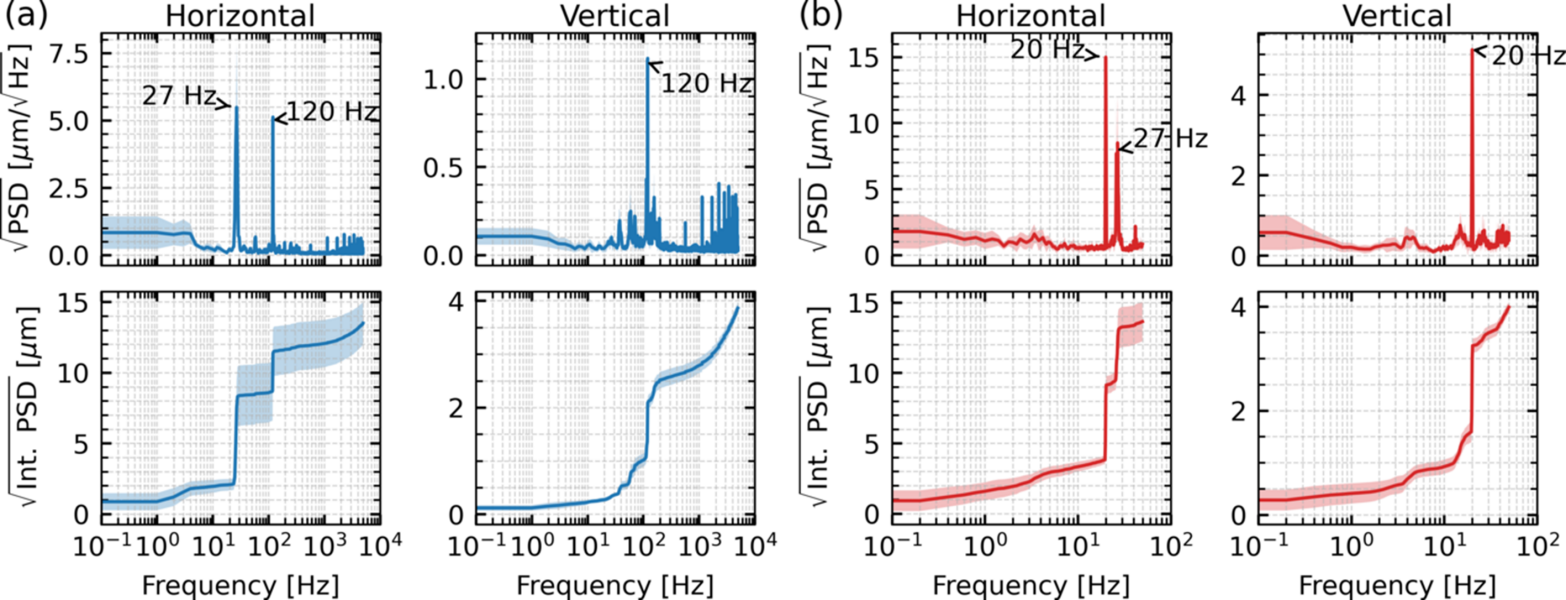
Square-root power spectral density (top) and square root of the integrated power spectral density (bottom) of baseline X-ray beam motion (without feedback) at the focusing point, measured by (*a*) XBPM-C (10 kHz sampling) and (*b*) the camera (100 frames s^^−1^^). Each curve represents the mean of ten acquisitions, with shaded bands indicating ±1σ (standard deviation).

We began by characterizing beam stability and evaluating the performance of the mechanical feedback. Data were collected at the focusing point and analyzed using power spectral density (PSD) and its integration. Fig. 2 ▸ shows the baseline vibration spectrum of the beam at the focusing point, measured by XBPM-C and the camera, under no-feedback conditions. The XBPM-C data shown in Fig. 2 ▸(*a*) reveal dominant peaks at 27 Hz and 120 Hz in the horizontal plane, and at 120 Hz in the vertical plane. For the camera, vibration analysis was performed on the centroid position of each image. The result, shown in Fig. 2 ▸(*b*), confirmed the presence of the same peaks. Because the camera operated at a frame rate of 100 Hz, the observable spectrum was limited to 50 Hz, causing the 120 Hz peak to alias as 20 Hz.

The integrated PSD represents the root-mean-square (RMS) vibration at the focusing point, which was approximately 13 µm in the horizontal plane and 4 µm in the vertical plane, with most of the contribution arising from the 27 Hz and 120 Hz peaks.

To estimate the origin of the dominant vibration components observed at the HXN focusing point, we compared beam motion at multiple locations along the system, starting from the electron beam source and progressing downstream through XBPM-FE, XBPM-B, and XBPM-C. The electron beam position and angle at the HXN source were reconstructed using upstream and downstream electron BPMs surrounding the undulator. The source position and angle (in either the horizontal or vertical plane) were calculated as 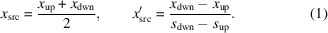
where *x*_up_ and *x*_dwn_ are the beam positions measured by the upstream and downstream electron BPMs, and *s*_up_ and *s*_dwn_ are their longitudinal locations in the storage ring.

**Figure 3:**
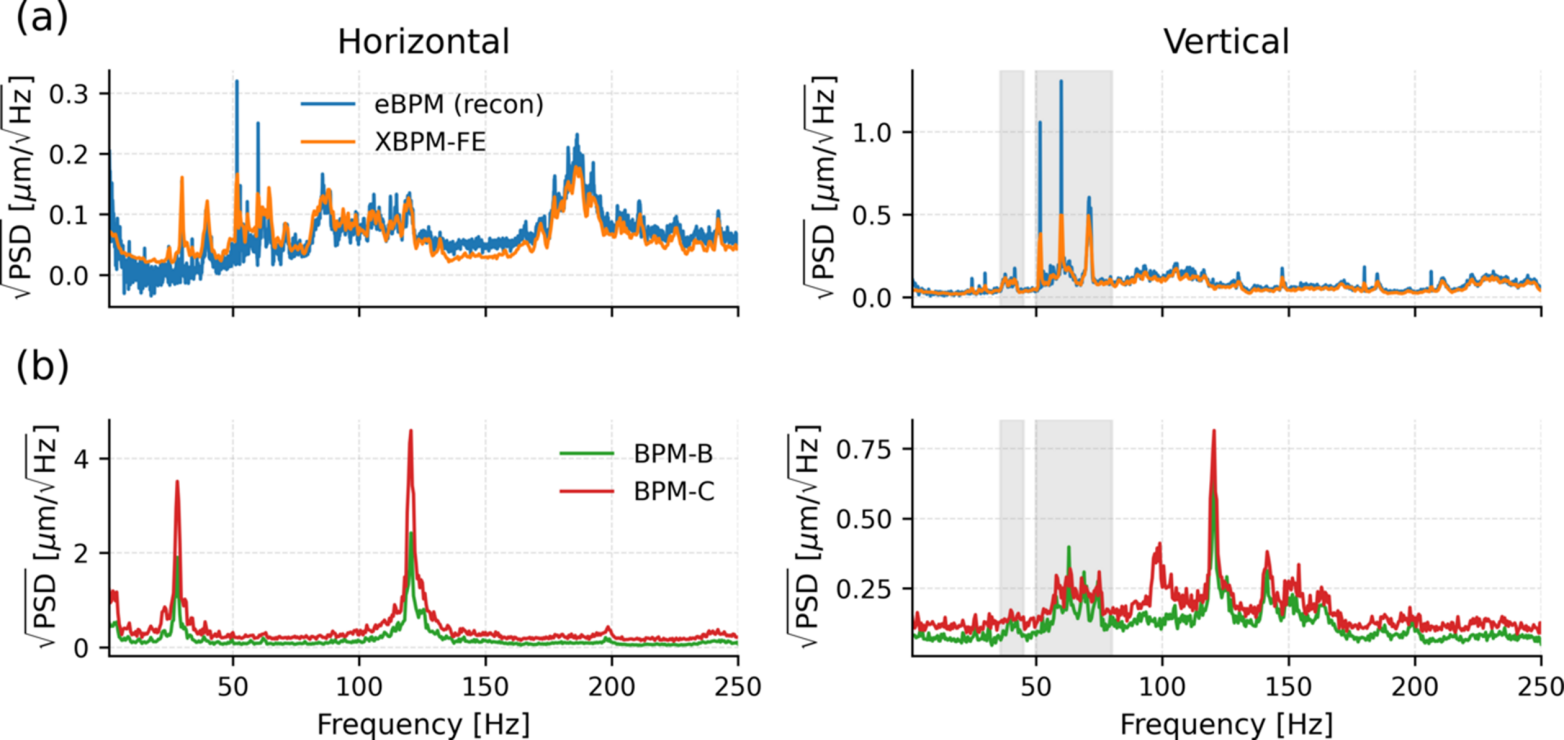
Square-root PSD comparison of beam motion measured at different locations along the HXN beamline under normal operating conditions. (*a*) Reconstructed electron beam motion at the source compared with XBPM-FE. (*b*) Downstream X-ray beam motion measured at XBPM-B and XBPM-C. Gray shaded regions indicate frequency ranges where electron-beam-related noise becomes visible in the XBPM-B and XBPM-C signals.

Using the reconstructed source parameters, the expected X-ray beam position at XBPM-FE was calculated as 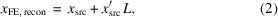
where *L* = 16 m is the distance from the source to XBPM-FE. Fig. 3 ▸(*a*) compares the PSD of the reconstructed electron beam motion with the measured XBPM-FE signal. The two spectra agree well and do not show pronounced narrowband peaks at 27 Hz or 120 Hz, in contrast to the spectra measured at XBPM-B and XBPM-C shown in Fig. 3 ▸(*b*). The small horizontal-plane peak observed at XBPM-FE occurs near 29 Hz, not corresponding to the dominant 27 Hz vibration observed downstream at XBPM-B and XBPM-C. The data shown in Fig. 3 ▸ were acquired under normal operating conditions with both the FOFB and the local mechanical feedback enabled.

These observations suggest that the dominant vibration components at 27 Hz and 120 Hz are not driven by the electron beam or introduced upstream of the optical elements but instead arise downstream of the front-end, most likely from beamline optics in Hutch A. The approximately twofold increase in amplitude from XBPM-B to XBPM-C is consistent with their relative distances from Hutch A (35 m and 65 m, respectively), supporting an optics-dominated origin.

In the vertical plane, vibrations related to electron beam are visible primarily below approximately 100 Hz, but their amplitudes observable at XBPM-B and XBPM-C are significantly smaller than the dominant 120 Hz peak under normal operating conditions with FOFB enabled, while in the horizontal plane the electron contribution is minimal. When FOFB is disabled, electron beam vibrations increase and can exceed the dominant peak in the vertical plane.

### 2.3. Impact on ptychography reconstructions

**Figure 4:**
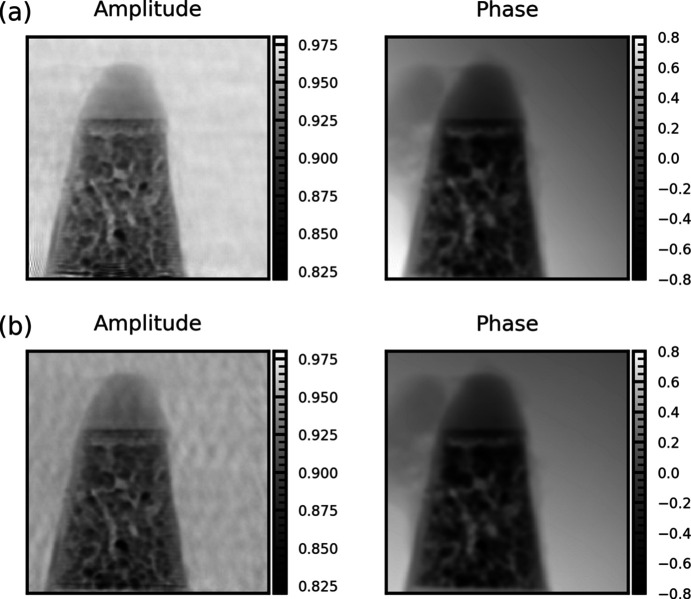
Ptychography amplitude and phase reconstructions of an Au nanoparticle superlattice acquired at dwell times of (*a*) 10 ms (100 Hz) and (*b*) 5 ms (200 Hz) under normal X-ray beam operating conditions with the local mechanical feedback enabled. The scan step size was 30 nm × 30 nm, and the grid size was 100 × 100.

To evaluate the effect of beam stability on image quality, we collected ptychography amplitude and phase images of an Au nanoparticle superlattice sample (Kim *et al.*, 2025 ▸). The measurements were performed at different scanning rates under normal operating conditions, with both the FOFB and local mechanical feedback enabled. Fig. 4 ▸ compares reconstructions acquired with dwell times of 10 ms (100 Hz) and 5 ms (200 Hz). The scan step size was 30 nm × 30 nm, and the grid size was 100 × 100. In the amplitude images, the slower scan (10 ms) averages out beam motion, producing a relatively uniform background, while the faster scan (5 ms) reveals background artifacts associated with beam vibration. These fluctuations may obscure weak-contrast features and reduce reconstruction fidelity. In contrast, the phase reconstructions show minimal differences between the two scan rates.

### 2.4. Investigation of the mechanical feedback performance

**Figure 5:**
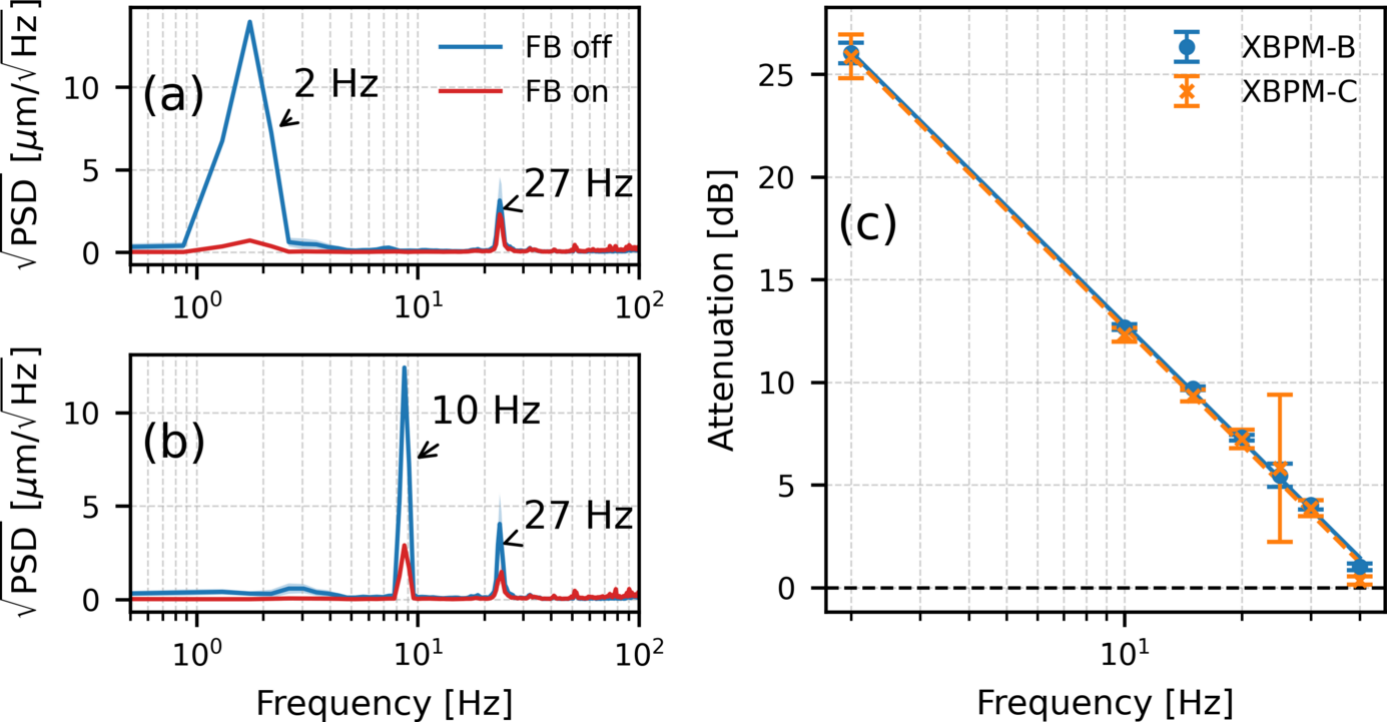
Mechanical feedback performance. Suppression of (*a*) a 2 Hz excitation and (*b*) a 10 Hz excitation, measured at XBPM-B. (*c*) Frequency-dependent attenuation measured at XBPM-B and XBPM-C, defined as 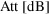 = 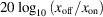.

As described earlier, HXN implements local mechanical feedback by using XBPM-B as the input signal to drive the piezo actuator of the monochromator. We characterized the performance of this system to evaluate its effectiveness in suppressing dominant vibration components. Sinusoidal excitations were applied to the X-ray beam at various frequencies, and the resulting X-ray beam motion at XBPM-B was compared with the feedback on and off, as shown in Fig. 5 ▸. With a 2 Hz excitation [Fig. 5 ▸(*a*)], the feedback substantially suppressed the vibration peak. However, as the excitation frequency was increased to 10 Hz [Fig. 5 ▸(*b*)], the suppression became less effective. The frequency-dependent attenuation observed at both XBPM-B and XBPM-C, expressed in dB, is summarized in Fig. 5 ▸(*c*). Similar attenuation trends are observed at the two locations, demonstrating consistent mechanical feedback performance along the beamline. The attenuation decreases with increasing frequency and approaches 0 dB near 40 Hz, showing that effective suppression is limited to frequencies below this range. The 27 Hz peak shows partial suppression but with lower attenuation than lower-frequency components, as it lies close to the feedback bandwidth. As a result, the mechanical feedback provides limited suppression of the dominant vibration peaks at 27 Hz.

These results motivated us to explore an alternative approach based on using the electron beam itself as the actuator for feedback, leveraging the high control bandwidth available through FOFB, which operates at approximately 400 Hz. In parallel, we are continuing to improve the local mechanical feedback through higher sampling rates and alternative control strategies, such as infinite impulse response and notch filtering, with machine-learning optimization; these developments will be reported separately. The present work focuses on investigating the feasibility and performance of electron-beam-based feedback for compensating X-ray beam vibrations at the beamline.

## 3. Developments and implementation

### 3.1. System architecture

**Figure 6:**
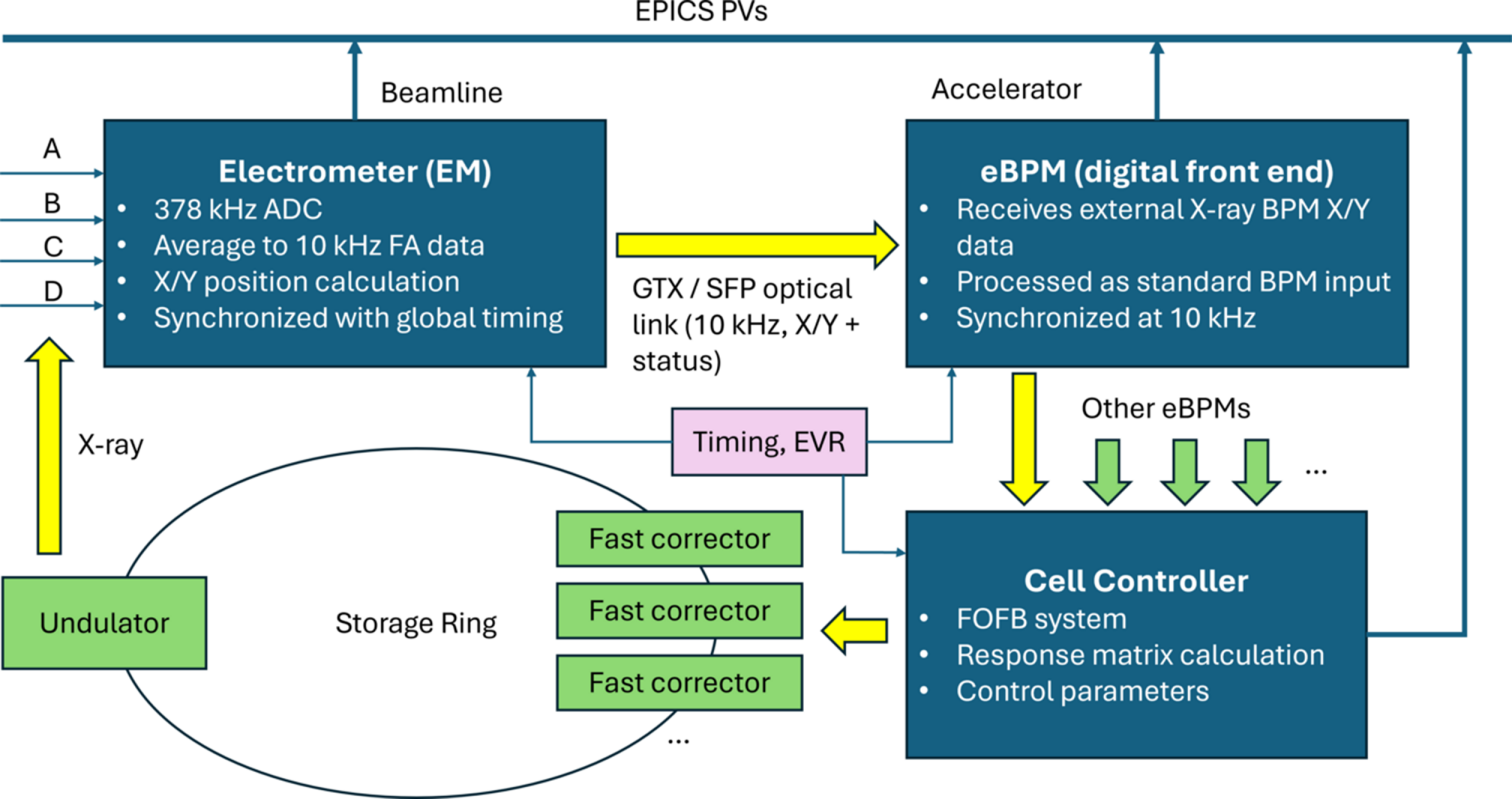
Schematic of the FXFB system integrated with the NSLS-II FOFB system.

In the remainder of this paper, the newly proposed electron-beam-based X-ray feedback is referred to as fast X-ray feedback (FXFB), to distinguish it from the existing mechanical feedback and the storage-ring FOFB. The FXFB system at NSLS-II is implemented as an FPGA-based extension of the existing storage-ring FOFB infrastructure (Tian *et al.*, 2015 ▸). The design integrates XBPMs into the accelerator feedback chain, enabling real-time use of photon beam position information while reusing established FOFB hardware, timing, and control architecture. Fig. 6 ▸ illustrates the system architecture and real-time data flow.

The XBPM signals are read out using an upgraded four-channel high-precision electrometer developed in-house at NSLS-II (Ha *et al.*, 2025 ▸). Each channel is digitized by a 20-bit ADC at a raw sampling rate of approximately 378 kHz derived from the accelerator timing system. The electrometer is implemented on a Xilinx Zynq-7030 system-on-chip, with signal preprocessing performed entirely in FPGA logic. Raw ADC samples are block-averaged to a fast-acquisition rate of 10 kHz. Horizontal and vertical X-ray beam positions are then computed with calibrated gains and offsets.

Beam position calculation, data framing, and transmission are executed in a fixed hardware pipeline synchronized to the NSLS-II timing system. The position data are aligned to the FOFB cycle using shared event codes, ensuring synchronous delivery with electron BPM data without reliance on software-based scheduling.

Computed X-ray beam position data are transmitted from the beamline to the accelerator feedback system using a dedicated SFP-based optical link implemented with FPGA GTX transceivers operating at a 5 Gbps line rate using 8b/10b encoding. Each data frame contains horizontal and vertical beam positions, auxiliary summed signals, status and validity flags.

X-ray beam position data are integrated into the existing FOFB architecture by reusing the in-house-developed electron BPM digital front-end (Ha *et al.*, 2011 ▸). The digital front-end receives X-ray beam position data bypassing the analog front-end used for electron BPMs and forwards them to the FOFB system in the standard BPM data format. This allows the FOFB Cell Controller to receive X-ray beam position information through the normal BPM data path and ensures compatibility with existing matrix-based feedback algorithms. Feedback computation, including matrix calculation and corrector command generation, is performed in the Cell Controller in the same manner as standard operation.

For diagnostics, beam position data used by the FXFB system are exposed through EPICS using process variables (PVs). The system also provides an internal buffer for triggered capture and storage of fast-acquisition beam position waveforms, which can be read out through EPICS waveform PVs for diagnostic analysis. Slow PVs provide access to status, timing, and health information to support commissioning and system characterization.

For the initial tests of FXFB performance, the implementation has focused on local orbit bump configurations to control source position and angle at the beamline, while the architecture already supports integration into the global FOFB loop.

### 3.2. Implementation of local orbit bump

**Figure 7:**
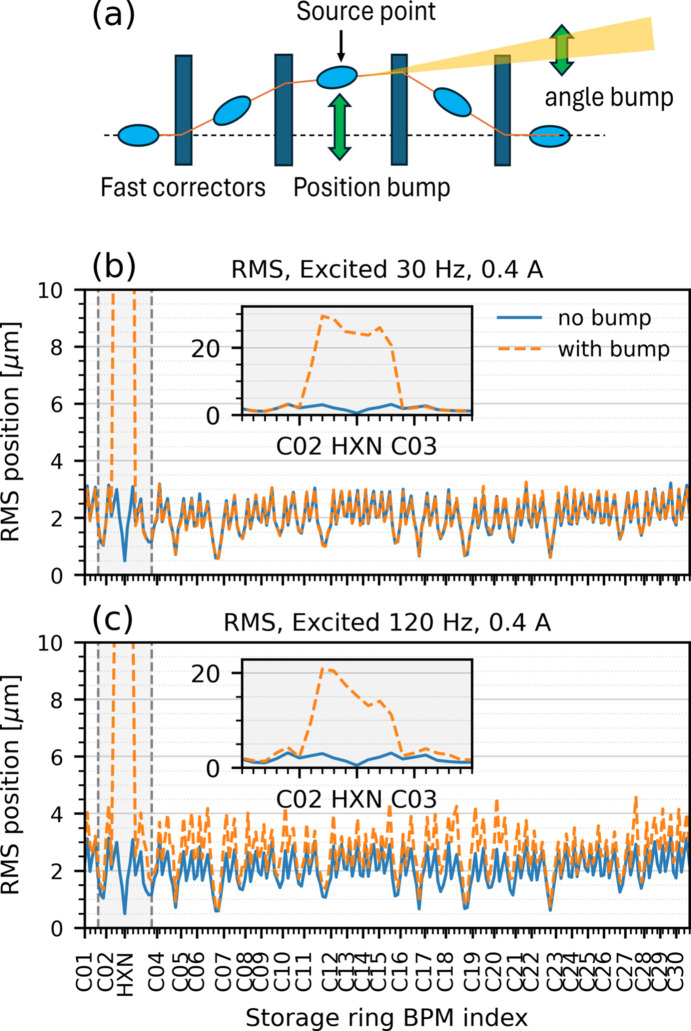
(*a*) Schematic of the local electron orbit bump using multiple fast correctors. RMS orbit for local bump excitation at (*b*) 30 Hz and (*c*) 120 Hz, respectively, with a corrector current of 0.4 A.

For the initial experimental tests, our primary objective was to evaluate the performance of FXFB in suppressing X-ray beam vibrations at the HXN beamline. To enable this study, global FOFB was disabled, and a local electron orbit bump was implemented around the HXN source. This method uses four or more fast corrector magnets to synchronously control the electron beam position and angle at a specified location, while preserving the beam orbit elsewhere, as illustrated schematically in Fig. 7 ▸(*a*). The local bump serves two purposes: first, it allows independent control of the source position and angle required for feedback studies; second, it reduces orbit leakage to the rest of the storage ring, thereby limiting unintended perturbations that could activate orbit interlocks and interrupt machine operation, ensuring safe conditions during these tests.

Fig. 7 ▸(*b*) shows a dynamic test of the local orbit bump in which a vertical position excitation was applied at 30 Hz. The excitation amplitude corresponds to a peak corrector current of 0.4 A, which is approximately 30% of the maximum output current of the fast corrector power supplies. Under this condition, the electron beam position at the HXN source reached an RMS amplitude of about 25 µm, while orbit leakage outside the bump region remained below 1 µm RMS. The measured orbit follows the expected bump shape, indicating that the relative corrector strengths remain well balanced at this frequency and that the local bump remains effectively closed under dynamic excitation in this range.

At higher excitation frequency, limitations of the present hardware become evident. Fig. 7 ▸(*c*) shows the corresponding result for a local bump excitation at 120 Hz using the same peak corrector current of 0.4 A. In this case, increased orbit leakage is observed at several locations around the ring, with additional RMS orbit deviations of up to approximately 2 µm above the baseline level. In addition, the orbit bump shape deviates from that observed at 30 Hz, indicating a degradation of the ideal bump closure. This behavior is likely attributed to effective slew-rate limitations of the fast corrector power supplies at higher frequency, arising from the inductive load of the corrector magnets and voltage compliance limits. As a result, the relative strengths among all the fast correctors become unbalanced at higher frequency, leading to distortion of the ideal bump configuration and increased orbit leakage.

These observations identify practical hardware limitations of the present implementation rather than a fundamental limitation of the local orbit bump technique itself. Future work will explore upgraded hardware, such as fast corrector power supplies, magnets, and associated vacuum chamber components. At the same time, the results provide strong motivation for unifying FXFB with FOFB, where the global corrector system can assist in distributing the correction effort and more effectively suppress residual orbit leakage.

## 4. Experimental results

### 4.1. X-ray beam stability measurements

With the local orbit bump established at the electron source, we tested the effect of FXFB on X-ray beam vibrations. We first used XBPM-B data as the input for the feedback system. Measurements showed that the X-ray beam at Hutch B responds strongly to the source angle but only weakly to the source position. This is likely because Hutch B is located far upstream from the focusing point. As a result, the source angle was selected as the actuator in this configuration, since using a position bump would require impractically large corrections to suppress the vibration observed at Hutch B.

**Figure 8:**
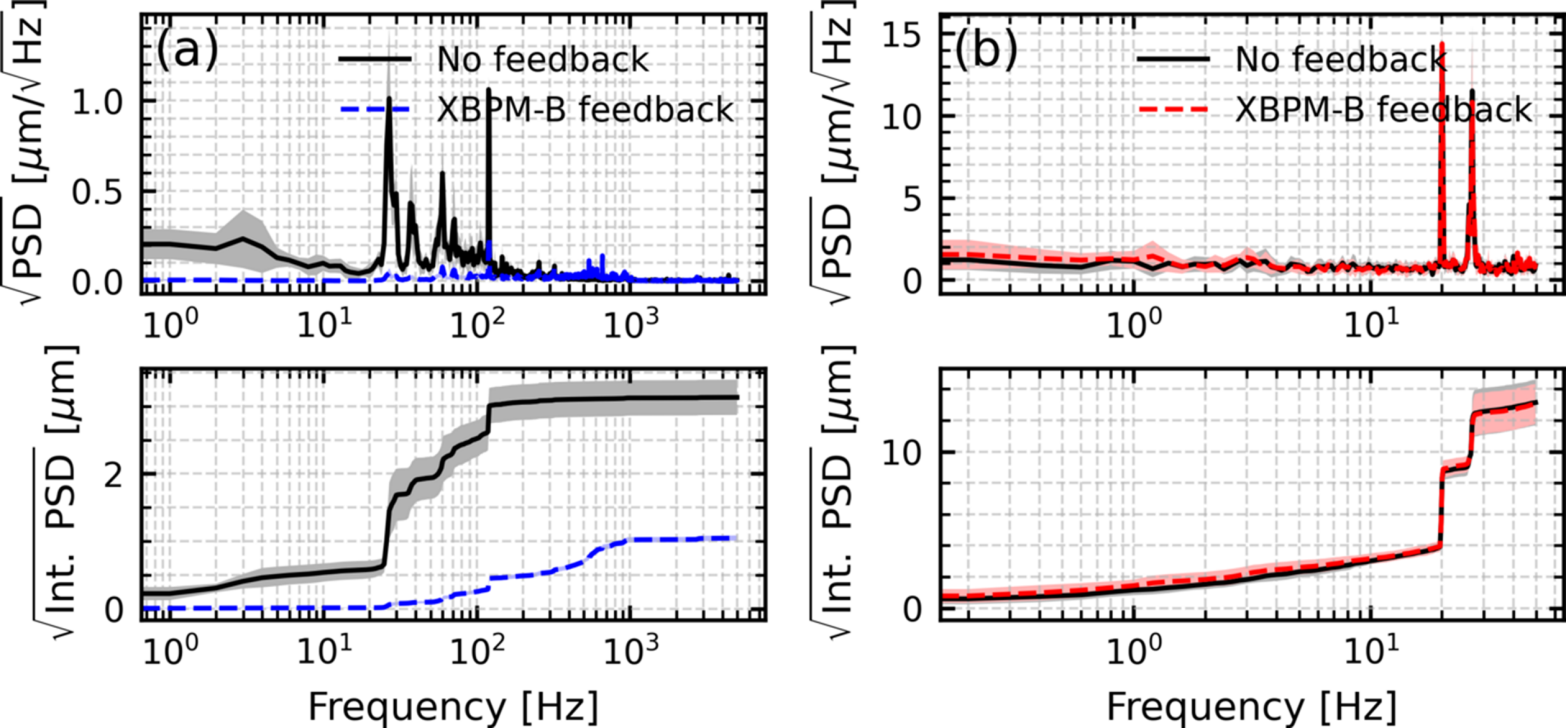
Square-root horizontal PSD (top) and square-root integrated PSD (bottom) of beam motion with and without feedback, using XBPM-B as the input signal. (*a*) Measured at XBPM-B. (*b*) Measured at the camera at the focusing point.

After optimizing the PID control parameters using *Badger* (Zhang *et al.*, 2022 ▸), a GUI-based tool that applies machine learning algorithms for online optimization, we compared the beam motion with feedback on and off. Fig. 8 ▸(*a*) shows that the feedback suppresses vibration peaks in the XBPM-B signal as expected. However, when monitoring the beam at the focusing point with the camera, no improvement in position stability was observed [Fig. 8 ▸(*b*)]. Separate measurements confirmed that the beam at the focus is primarily sensitive to the source position and only slightly to the source angle. Therefore, using XBPM-B with the source angle as the actuator likely reduced beam divergence fluctuations rather than directly improving position stability at the focusing point.

**Figure 9:**
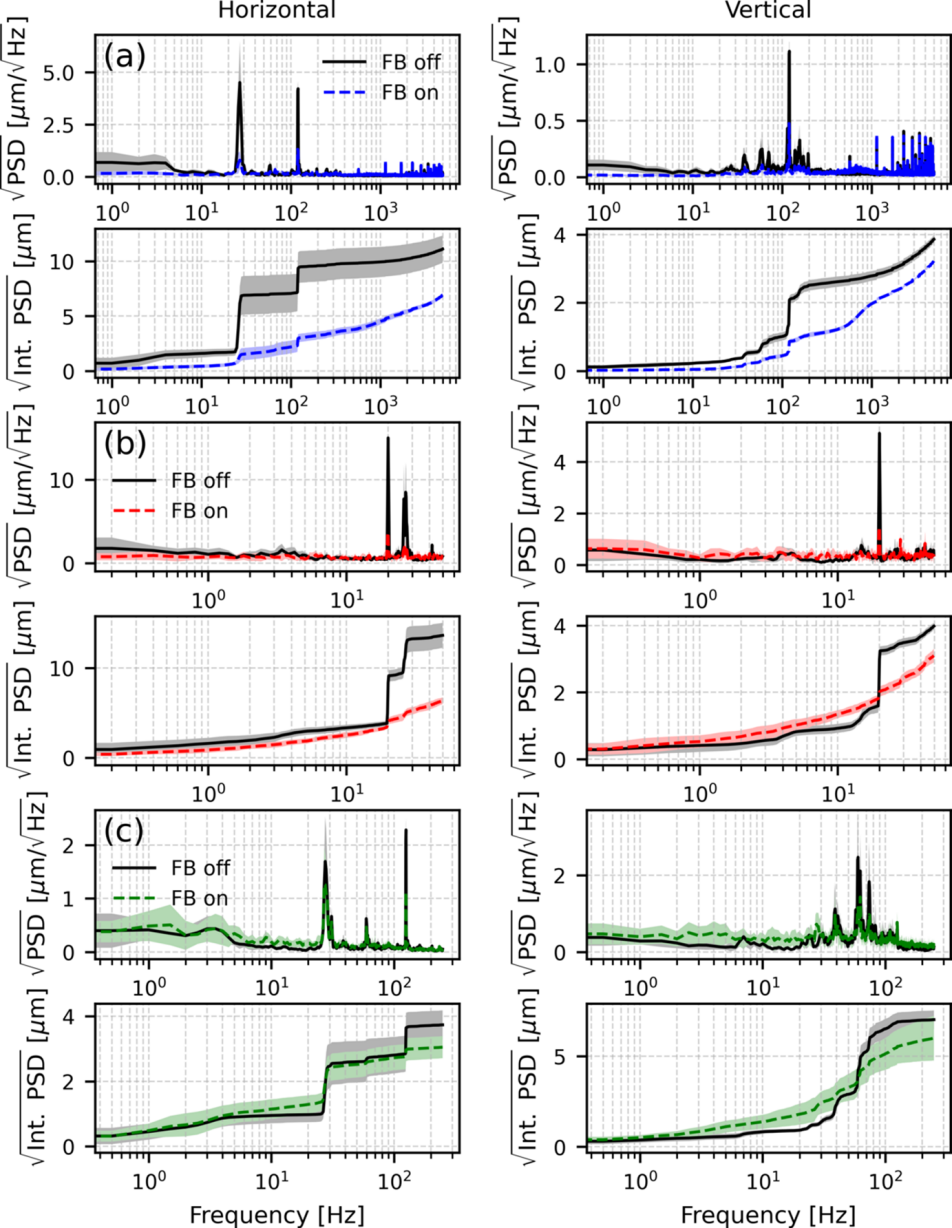
Horizontal and vertical beam motion shown as square-root PSD (top) and square-root integrated PSD (bottom) with and without FXFB, using XBPM-C as the input signal. Measurements are shown at (*a*) XBPM-C, (*b*) the camera at the focusing point, and (*c*) XBPM-B.

Next, we switched to using XBPM-C as the input for the FXFB. XBPM-C is located adjacent to the focusing point and, like the camera, responds primarily to the source position. After optimizing the PID control using the position bump as the actuator, we compared the vibration data measured by XBPM-C and the camera. Fig. 9 ▸ shows the noise spectra with and without feedback. The dominant peaks at 27 Hz and 120 Hz were suppressed by more than 80%, resulting in a significant improvement in RMS position stability at the focusing point.

Fig. 9 ▸(*c*) shows the square-root PSD measured at XBPM-B from the same experiment, with FXFB on and off. In the horizontal plane, partial suppression of the 120 Hz peak (approximately 50%) is observed when the FXFB is enabled, which is smaller than the suppression achieved at XBPM-C. In contrast, the 27 Hz component shows little suppression at XBPM-B despite being strongly reduced at XBPM-C. The remaining 27 Hz peak at XBPM-B reflects its higher sensitivity to source angle, suggesting that incorporating an angle bump could further improve suppression of divergence-related X-ray motion in addition to position stabilization. In the vertical plane, since these measurements were performed with the global FOFB disabled, electron beam motion around 40–80 Hz dominates the 120 Hz peak at XBPM-B (but not at XBPM-C, suggesting that the baseline electron-beam vibration is primarily angle related); consequently, only a small improvement is observed at XBPM-B when using XBPM-C as the feedback input.

### 4.2. Impact on ptychography reconstructions

**Figure 10:**
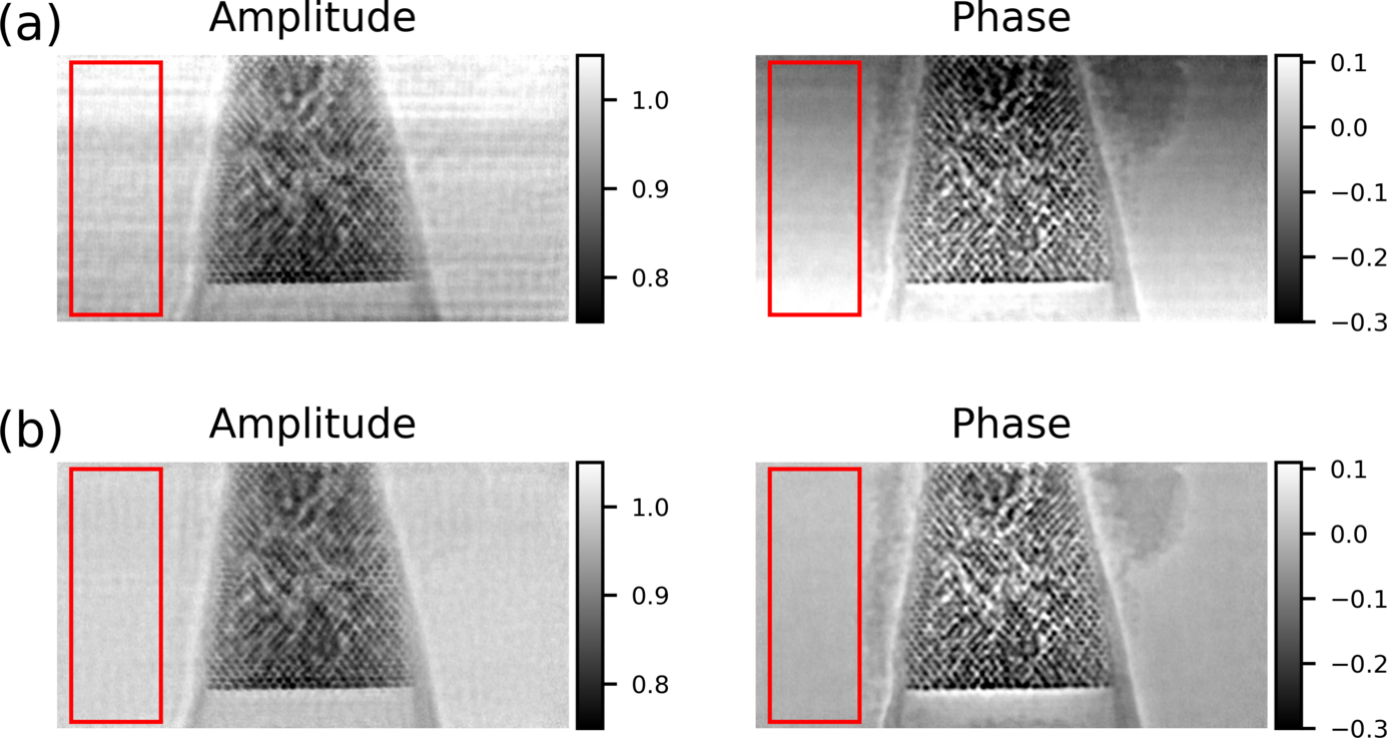
Ptychography reconstructions of an Au nanoparticle superlattice acquired at a dwell time of 3.2 ms, showing (*a*) FXFB off and (*b*) FXFB on, using XBPM-C as the input. Both amplitude and phase images are shown, with the red box indicating the ROI used for analysis. The scan step size was 20 nm × 20 nm with a 200 × 100 grid.

Finally, to confirm the practical benefits of FXFB for HXN, we performed ptychography scans as a benchmark. Fig. 10 ▸ compares amplitude and phase reconstructions obtained with the source feedback disabled and enabled, using XBPM-C as the input signal. The scan step size was 20 nm × 20 nm with a grid of 200 × 100 points. The dwell time of 3.2 ms (315 Hz) was chosen to emphasize the effect of high-frequency noise such as the 120 Hz vibration peak. Without feedback, periodic background patterns caused by X-ray beam vibration overlap with the sample features, potentially introducing errors in weak-contrast regions. With feedback enabled, these background patterns are strongly suppressed, yielding a much cleaner and more uniform image.

**Figure 11:**
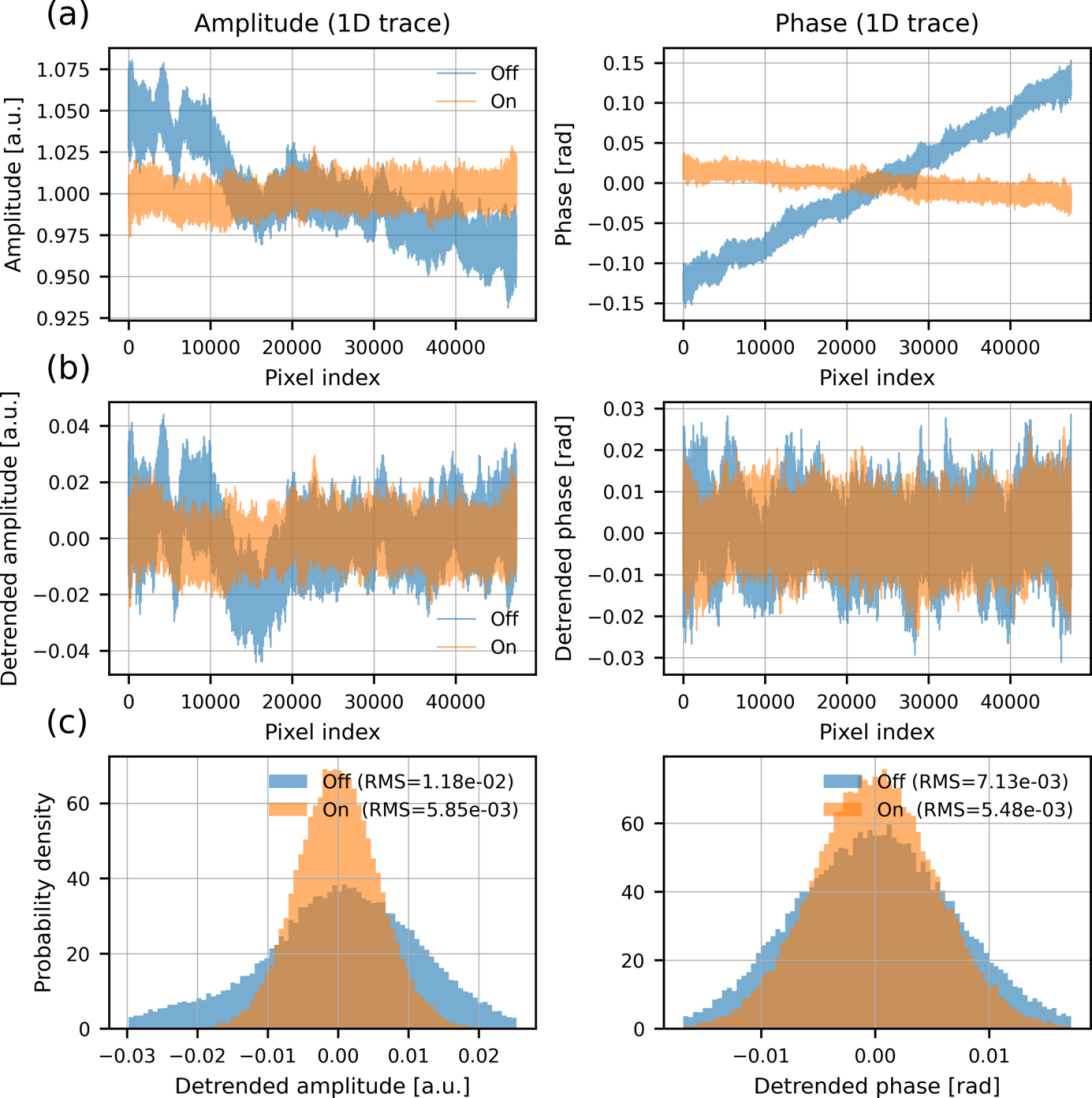
Analysis of background fluctuations in the ptychography reconstructions. (*a*) One-dimensional traces of amplitude and phase extracted from the ROI (raw data). (*b*) Corresponding traces after linear detrending. (*c*) Histograms of the detrended amplitude and phase values.

To quantify the improvement observed in Fig. 10 ▸, we analyzed background fluctuations in the reconstructed images using the region of interest (ROI) indicated by the red boxes. Fig. 11 ▸ summarizes this analysis using one-dimensional traces extracted from the ROI.

Fig. 11 ▸(*a*) shows the raw amplitude and phase values plotted as a function of pixel index for the FXFB-off and FXFB-on cases. Without feedback, both amplitude and phase exhibit a clear spatial gradient across the ROI, consistent with slow X-ray beam drift during the scan. When the FXFB is enabled, this slow drift is substantially reduced, resulting in a flatter baseline in both channels.

Such gradients can often be partially corrected during image post-processing and have a small effect on overall image appearance. To focus on fluctuations that directly affect reconstruction quality, the images were detrended by subtracting a best-fit linear plane from the ROI. Fig. 11 ▸(*b*) shows one-dimensional traces of amplitude and phase after detrending. With feedback enabled, the residual fluctuations are smaller, indicating reduced point-to-point variation.

The statistical distributions of these detrended fluctuations are shown in Fig. 11 ▸(*c*). When feedback is applied, the distributions become noticeably narrower. For the amplitude, the RMS fluctuation decreases from 1.18 × 10^−2^ a.u. without feedback to 5.85 × 10^−3^ a.u. with feedback enabled, corresponding to an improvement of approximately 50%. For the phase, the RMS fluctuation decreases from 7.13 × 10^−3^ rad to 5.48 × 10^−3^ rad, corresponding to an improvement of approximately 23%. These results confirm that FXFB not only suppresses large-scale slow drift but also reduces local amplitude and phase fluctuations that degrade ptychographic reconstruction quality.

To determine whether the observed improvement is associated with suppression of the dominant vibration components at 27 Hz and 120 Hz, we analyzed Fourier spectra of background fluctuations extracted from the ptychography reconstructions with and without FXFB.

**Figure 12:**
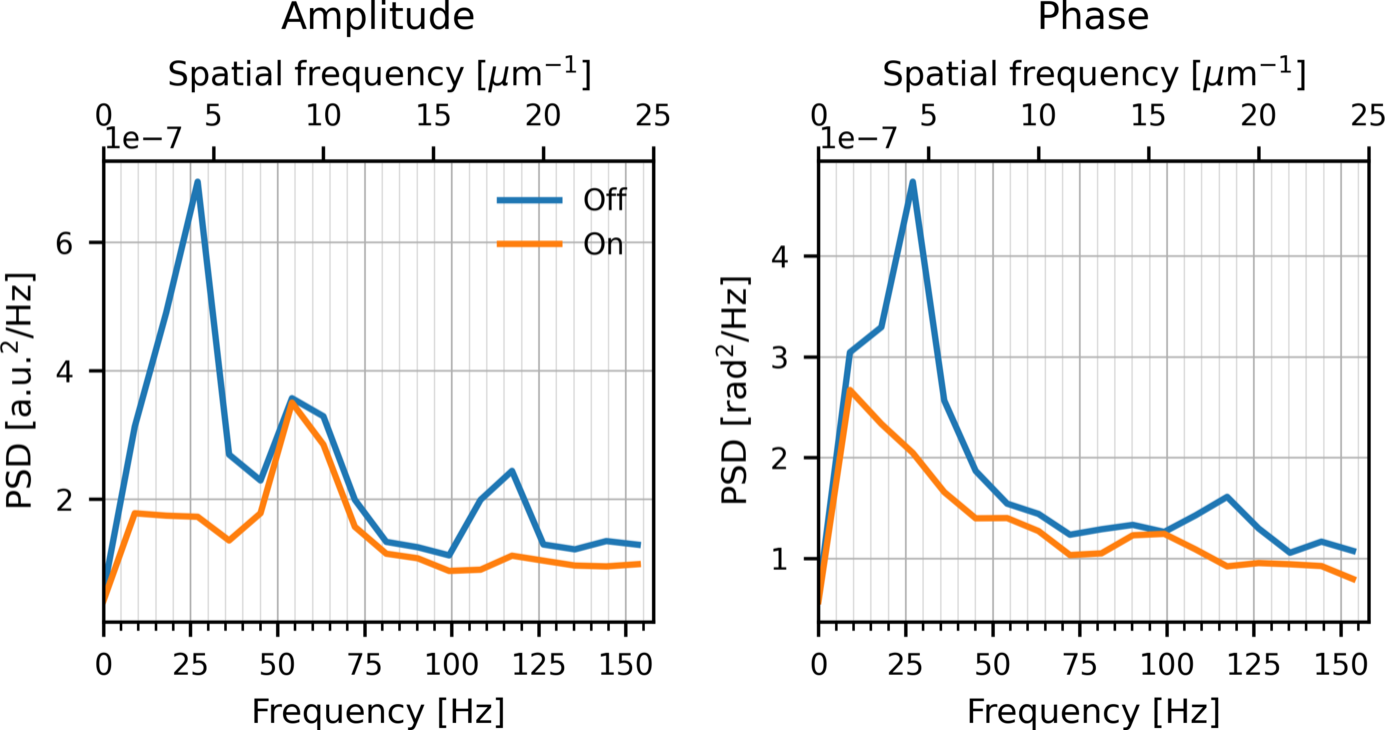
Power spectral density (PSD) of background fluctuations extracted from the ptychography reconstructions along the fast-scan (horizontal) direction, showing amplitude (left) and phase (right) for FXFB off and on.

The PSD was calculated for each row and then averaged over all rows. The scan follows a zigzag raster pattern, with the horizontal direction corresponding to the fast scan; therefore, the Fourier transform was performed only along this direction. The scan step size was 20 nm with a dwell time of 3.2 ms per point (315 Hz), and the reconstructed images have a real-space pixel size of 5.38 nm, corresponding to an effective upsampling factor of approximately 3.7 image pixels per scan point. After mapping the spatial sampling to the time domain, the resulting spectra are shown in Fig. 12 ▸.

As shown in Fig. 12 ▸, with FXFB disabled, clear peaks appear near 27 Hz and 120 Hz in both the amplitude and phase spectra, consistent with the dominant X-ray vibration components. When FXFB is enabled, both peaks are strongly suppressed. A residual peak near 60 Hz remains visible in the amplitude spectrum and is not significantly affected by the feedback, likely reflecting electronic noise associated with the power-line frequency within the detector electronics. These results confirm that the improvement in ptychography image quality directly corresponds to suppression of the dominant X-ray beam vibrations observed in the XBPM measurements.

**Figure 13:**
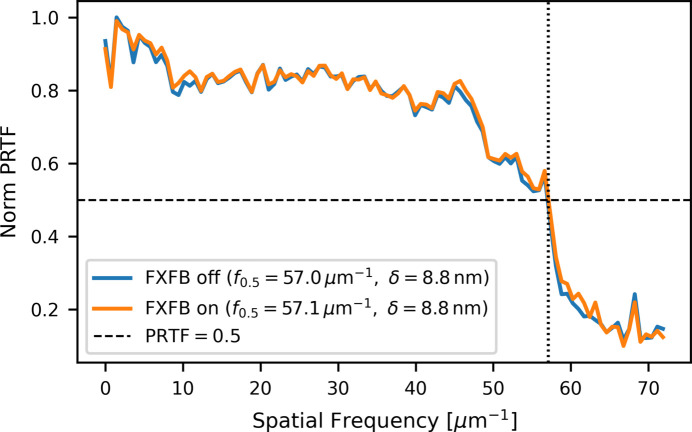
Phase-retrieval transfer function (PRTF) for FXFB off and on. Both cases show the same resolution of 8.8 nm at the 50% PRTF criterion.

Finally, the phase-retrieval transfer function (PRTF) was also evaluated to compare the achieved spatial resolution with and without feedback. PRTF was evaluated by comparing radially averaged calculated and measured diffraction intensities as a function of spatial frequency, following practical implementations used in ptychography (Chapman *et al.*, 2006 ▸; Wilke *et al.*, 2013 ▸). Fig. 13 ▸ shows the PRTF curves for the FXFB off and on cases, which exhibit nearly identical behavior over the full spatial frequency range.

The spatial resolution was estimated using the 50% PRTF criterion and defined using the standard half-period convention,
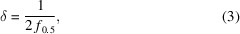
where *f*_0.5_ is the spatial frequency at which the normalized PRTF drops to 0.5. Using this definition, both cases yield a resolution of approximately 8.8 nm, indicating no significant change in the achieved resolution when the feedback is enabled.

These results show that the improvement provided by the FXFB is reflected in reduced image fluctuations rather than an improvement in nominal spatial resolution. The main benefit is the suppression of vibration-induced artifacts that can distort image patterns, particularly in low-contrast features, and lead to misinterpretation of reconstructed sample structures.

## 5. Challenges and future plans

**Figure 14:**
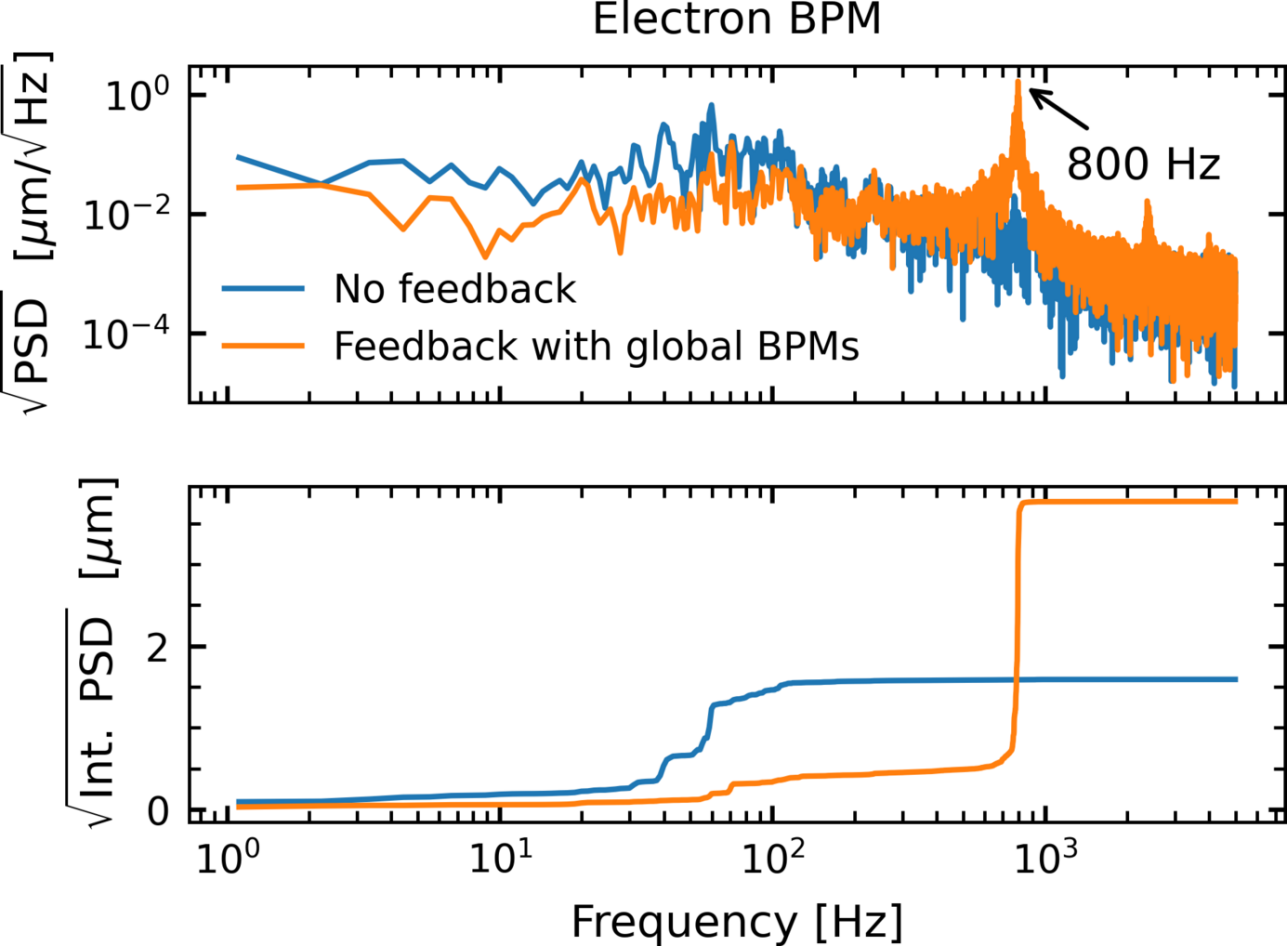
Example of electron beam position spectra showing an 800 Hz oscillation when local and global feedback loops run simultaneously.

Beam stabilization using FXFB has been shown to enhance performance at the HXN beamline. The next step is to integrate this capability into routine operation, which presents several challenges. To date, tests have been performed with FXFB operating alone, while FOFB was disabled. When both systems were enabled simultaneously but operated as separate loops, a strong oscillation appeared near 800 Hz, as shown in Fig. 14 ▸, indicating interference between them.

A key motivation for integrating X-ray data into the FOFB is to unify the electron-based global feedback and the X-ray feedback within a single control loop, thereby enabling coordinated correction of both electron and photon beam motion. Such an approach would prevent conflicts between independent feedback loops and promote stable, cooperative control across both domains. Development of this unified scheme is in progress and will be reported in future studies.

In parallel with control-system integration, local-bump experiments highlight hardware-related limitations that become apparent at higher excitation frequencies. In addition, during FXFB experiments, relatively large corrector currents are required to suppress observed beam motion, further indicating limited performance margin in the present hardware. Future work will therefore also explore upgrades to the fast corrector hardware, including power supplies, magnets, and associated vacuum chamber components, to improve overall performance and robustness under dynamic operation. Such hardware improvements are expected to complement the unified feedback architecture by extending the usable operating range and reducing sensitivity to high-frequency limitations.

Another ongoing effort focuses on combining signals from XBPM-B and XBPM-C to suppress X-ray motion in both position and angle. This multi-input approach also has the potential to reduce beam divergence fluctuations at the focusing point and further improve image quality.

An additional challenge arises from local events at HXN, such as signal loss due to shutter closures or X-ray beam realignment, which can impact global stability in the storage ring. To mitigate this, we plan to develop an active interlock system capable of detecting anomalies at the beamline and temporarily returning control to the global electron orbit feedback. This safeguard will be essential before this feedback can be integrated into routine operation.

## 6. Summary

We have developed and experimentally evaluated an electron-beam-based feedback scheme, referred to as FXFB, to explore its potential and current limitations for enhancing X-ray beam stability at the HXN beamline of NSLS-II. Beam characterization revealed dominant vibration peaks at 27 Hz and 120 Hz that could not be effectively mitigated by the existing mechanical feedback system, which only has a bandwidth on the order of several tens of hertz. To overcome this limitation, we introduced the concept of using the electron beam itself as the actuator for feedback. This was achieved by integrating XBPM signals into the existing FOFB system through a dedicated electrometer and implementing a local orbit bump around the source of HXN. Using XBPM-C as the input, the feedback suppressed vibration peaks by more than 80% and significantly improved position stability at the focusing point. Benchmark ptychography measurements confirmed these improvements, showing reduced background fluctuations and enhanced image stability in both amplitude and phase. These results demonstrate the feasibility of electron-source feedback as an effective approach for enhancing X-ray beam stability. This work provides guidance for future improvements in hardware, control integration, and system robustness based on the observed capabilities and limitations of FXFB. The results also motivate continued work toward unified global–local integration, multi-input feedback configurations, and active interlocks as key developments for achieving reliable routine operation at NSLS-II and future light source facilities.

## Data Availability

Upon request.
